# 

**Published:** 1889-04

**Authors:** 


### Dental Meetings.

The Kansas State Dental Association will hold its next annual meeting at Topeka, Kansas, commencing Tuesday, April 30th, 1889, and continuing four days. C. B. GUNN, Sec.

The Dental Society of the State of New York will hold its next regular annual meeting at Albany, Wednesday and Thursday, May 8th and 9th, 1889.

Members of the profession are cordially invited to be present and participate in the discussion of papers.

G. E. CURTIS, Correspondent.

The twenty-first annual meeting of the Dental Society of the State of New York will be held in the common council chambers in the city of Albany, N. Y., on Wednesday, May 8th, 1889.

The Board of Censors will meet on the day next preceding for the examination of candidates for the degree of M. D. S.

MYRON D. JEWELL, Sec.

The annual meeting of the Iowa State Dental Society will be held at Desmoines commencing May 7, 1889, and gives promise of being an interesting meeting. Dr. W. X. Sudduth, Philadelphia, has promised to be present and give a lantern exhibit and lecture on Pulp lesions.

F. R. ROSS,

Cedar Rapids. *Chairman Executive Committee.*

The annual meeting of the Mississippi Dental Association will be held at Vicksburg, Miss., commencing on Tuesday, May 21, 1889.

E. E. SPINKS,

Meridian, Miss. *Cor. Secretary.*

The tenth annual meeting of the Nebraska State Dental Society will be held in Wahoo, on the Third Tuesday in May, 1889, continuing three days.

From the present outlook this promises to be the best meeting in the history of the society. Several men of prominence in the profession from abroad will be with us.

J. J. WILLEY,

Wahoo, Neb. *Cor. Secretary.*

The Kansas State Dental Association will hold its next annual meeting at Topeka, Kansas, commencing on Tuesday, April 30, 1889, and continuing four days.

C. B. GUNN,

Leavenworth, Kan. *Secretary.*

The American Medical Association will hold its fortieth annual meeting at Newport, R. I., June 25, 26, 27, and 28, 1889. Intendiug exhibitors should address Dr. Charles A. Brackett, Newport, R. I., chairman sub-committee upon exhibits.

HORATIO R. STORER, M.D.,

*Chairman Committee of Arrangements.*

The Illinois State Board of Dental Examiners will meet at the New Council, in Quincy, on Monday, May 13, 1889, at 10 o'clock A. M., at which time candidates for examination must be promptly on hand.

C. R. E. KOCH, Secty.

The Seventh District Dental Society of the State of New York, will hold its Twenty-first Annual Convention in the city of Rochester, on the last Tuesday of April, and will continue through Wednesday the 1st of May.

The special Committee are arranging for clinics which will give the convention unusual interest.

The papers to be read will be of great practical value. Members of the profession are cordially invited to be present.

For further information address, The Recording Secretary.

Wm. Frank Arnold, D.D.S.,
235 East Main Street,
Rochester, N. Y.

---

MINNESOTA STATE DENTAL ASSOCIATION, 1889.

The Sixth Annual Meeting will be held in Duluth, Wednesday, Thursday and Friday, July 10, 11 and 12.

We have assurance, that reduced rates will be secured at hotels and railroads.

Duluth is most pleasantly situated for a summer meeting, and everything points to a profitable and enjoyable occasion. The committee cordially invite your presence.

The following list of subjects will be discussed, and in order that they may be presented in the most thorough manner, each subject will be placed in the hands of some member to edit and bring before the meeting, and our friends everywhere are solicited to assist us by contributions on any or all of these subjects, of manuscript, clinics, models, slides, drawings or appliances. (Instruments and appliances will be given ample room, and exhibited by themselves.)

Subjects: Implantation, electro-therapeutics, irregularities—causes and corrections; art in its relation to dentistry, comparative anatomy of the teeth, histology and pathology, nitrous oxide—anæsthetics; amalgams—varieties and methods; erosion and abrasion. Contributions upon any other subjects will be gladly received.

Address all communications to

Dr. L. D. Leonard,
Syndicate Block,
Minneapolis, Minn.

